# Astrocyte-Mediated Neuronal Synchronization Properties Revealed by False Gliotransmitter Release

**DOI:** 10.1523/JNEUROSCI.2761-16.2017

**Published:** 2017-10-11

**Authors:** Tiina M. Pirttimaki, Robert E. Sims, Gregory Saunders, Serena A. Antonio, Neela Krushna Codadu, H. Rheinallt Parri

**Affiliations:** School of Life and Health Sciences, Aston University, Birmingham B4 7ET, United Kingdom

**Keywords:** cortex, EAAT, glia, thalamus

## Abstract

Astrocytes spontaneously release glutamate (Glut) as a gliotransmitter (GT), resulting in the generation of extrasynaptic NMDAR-mediated slow inward currents (SICs) in neighboring neurons, which can increase local neuronal excitability. However, there is a deficit in our knowledge of the factors that control spontaneous astrocyte GT release and the extent of its influence. We found that, in rat brain slices, increasing the supply of the physiological transmitter Glut increased the frequency and signaling charge of SICs over an extended period. This phenomenon was replicated by exogenous preexposure to the amino acid D-aspartate (D-Asp). Using D-Asp as a “false” GT, we determined the extent of local neuron excitation by GT release in ventrobasal thalamus, CA1 hippocampus, and somatosensory cortex. By analyzing synchronized neuronal NMDAR-mediated excitation, we found that the properties of the excitation were conserved in different brain areas. In the three areas, astrocyte-derived GT release synchronized groups of neurons at distances of >;200 μm. Individual neurons participated in more than one synchronized population, indicating that individual neurons can be excited by more than one astrocyte and that individual astrocytes may determine a neuron's synchronized network. The results confirm that astrocytes can act as excitatory nodes that can influence neurons over a significant range in a number of brain regions. Our findings further suggest that chronic elevation of ambient Glut levels can lead to increased GT Glut release, which may be relevant in some pathological states.

**SIGNIFICANCE STATEMENT** Astrocytes spontaneously release glutamate (Glut) and other gliotransmitters (GTs) that can modify neuronal activity. Exposing brain slices to Glut and D-aspartate (D-Asp) before recording resulted in an increase in frequency of GT-mediated astrocyte–neuron signaling. Using D-Asp, it was possible to investigate the effects of specific GT release at neuronal NMDARs. Calcium imaging showed synchronized activity in groups of neurons in cortex, hippocampus, and thalamus. The size of these populations was similar in all areas and some neurons were involved in more than one synchronous group. The findings show that GT release is supply dependent and that the properties of the signaling and activated networks are largely conserved between different brain areas.

## Introduction

Astrocytes interact with neurons to sustain synaptic and network function, which underlies the emergence of activities that define the functioning brain (Lörincz et al., 2009, Poskanzer and Yuste, 2011, Lee et al., 2014, Oliveira et al., 2015). In addition to roles such as the uptake of released neurotransmitters, potassium homeostasis, and metabolic support, astrocytes also interact dynamically with neurons by the release of chemical messengers termed gliotransmitters (GTs) (Araque et al., 2014). There is ongoing and rigorous debate on the specific mechanisms of release of these GTs (Fiacco et al., 2007, Fujita et al., 2014), but there is consensus that GTs have a range of important physiological roles (Sloan and Barres, 2014).

A major way that GTs affect brain function is by modulating synaptic transmission, most notably in long-term potentiation (Jourdain et al., 2007, Henneberger et al., 2010) and long-term depression (Min and Nevian, 2012). Analysis of the anatomical and experimental evidence of astrocyte enwrapping of presynaptic and postsynaptic elements by astrocyte processes led to the “tripartite synapse” hypothesis (Araque et al., 1999). This hypothesis posits that astrocytes sense and respond to synaptic transmission and then release GTs that form feedback to the synapse and modify its function. Later, from mainly anatomical and morphological structure evidence came the concept of “synaptic islands,” in which a single astrocyte contacts many synapses on different neurons and so can exert control on these groups of spatially localized synapses (Halassa et al., 2007).

A defining feature of the functioning nervous system is synchronized activity. In most described cases, synchronized activity in a neuronal population is generated by action-potential-mediated neurotransmitter release (Buzsáki and Draguhn, 2004). In this way, glutamatergic or GABAaergic transmission can synchronize neurons locally (Allen and Monyer, 2015) and over long distances (Buzsáki and Wang, 2012). Synchronization can, however, also be sustained by nonsynaptic mechanisms; for example, via gap junction electrical coupling (Hughes et al., 2011). A distinct mechanism by which astrocytes can exert synchronizing control is through the phasic release of GTs acting at extrasynaptic receptors and generating slow inward currents (SICs). Synchronized SICs have been detected in paired recordings from neurons in hippocampus (Angulo et al., 2004, Fellin et al., 2004), thalamus (Pirttimaki et al., 2011) and nucleus accumbens (D'Ascenzo et al., 2007) and synchronized neuronal groups have been observed by inducing astrocyte activation in calcium-imaging experiments (Fellin et al., 2004).

Investigating the effects of GT release requires that neuronal activity is accounted for and consideration is given to the method used to elicit GT release; for example, the use of “nonphysiological” stimulation such as photolytic calcium release has been questioned (Wang et al., 2013) and exogenously applied astrocytic receptor agonists may also activate neuronal receptors. Physiologically, GT release occurs spontaneously (Parri et al., 2001); therefore, perhaps the most appropriate approach to determining GT effects is to study the emergence of these events. However, this is usually compounded by the fact that these events are relatively rare when recording from single neurons, approximately every 10 min in the ventrobasal (VB) thalamus and every 30 min in the nucleus accumbens (D'Ascenzo et al., 2007). Determining the fundamental relationships between astrocytes and neurons is important and could help to illuminate our understanding of the control and modulation of neuronal networks. For example, the spatial extent of this excitatory influence is likely underestimated. Using patch-clamp recordings, it is difficult to ascertain the number of neurons affected by a single event and SICs may activate the dendrites of neurons with cell bodies that are relatively distant.

In this study, we developed a gain-of-function model that exploits the fact that astrocytes take up neurotransmitter amino acids via excitatory amino acid transporters (EAATs) and then releases them phasically. Using D-aspartate (D-Asp) as a false GT, we investigated the spatial and temporal properties of SIC-mediated neuronal activation in hippocampus, sensory thalamus, and somatosensory cortex.

## Materials and Methods

### 

#### 

##### Experiments.

All experiments were approved by local ethical review and in accordance with the UK Animals Scientific Procedures Act of 1986 and current EU legislation. Both male and female Wistar rats (postnatal day 10 [P10]–P16 and P19–P21) were used in the study and killed by isoflurane overdose followed by cervical dislocation.

##### Slice preparation and maintaining solutions.

Slices of rat brain were prepared as described previously (Parri and Crunelli, 2001). Briefly, after removal from the skull, the brain was glued with cyanoacrylate adhesive to a metal block and submerged in the bath of a Microm MV tissue slicer. The bathing solution contained the following (in mm): NaCl 120, NaHCO_3_ 16, KCl 1, KH_2_PO_4_ 1.25, MgSO_4_ 5, CaCl_2_ 2, and glucose 10 and was maintained at <5°C. Thalamic slices were cut in the horizontal plane and hippocampal and somatosensory cortical slices in the coronal plane (all at 300 μm); all slices were stored in a 95% O_2_, 5% CO_2_ bubbled solution of identical composition at room temperature.

After a 1 h recovery period, experiments were performed in an artificial CSF (aCSF) solution containing the following (in mm): NaCl 120, NaHCO_3_ 16 or 25, KCl 2, KH_2_PO_4_ 1.25, MgSO_4_ 1, CaCl_2_ 2, and glucose 10 at room temperature (20–24°C) unless otherwise stated. Chemicals were obtained from Sigma-Aldrich except D-AP5, which was obtained from Ascent Scientific.

##### Fluorescence imaging.

Slices were loaded with Fluo-4 AM (Invitrogen) to monitor the dynamics of neuronal intracellular calcium ([Ca^2+^]_i_) responses. Slices were first treated with 1 mm Fluo-4 in DMSO for 3 min and later placed in standard loading solution and conditions (Aguado et al., 2002) with 1 μm sulforhodamine 101 (SR101) (Kafitz et al., 2008). Neuronal [Ca^2+^]_i_ increases were identified by their relatively larger signal diameter, faster and shorter [Ca^2+^]_i_ elevations compared with smaller-diameter astrocytes, and lack of SR101 loading. During the 2 min experiment acquisition time, neuronal responses were significantly more common in neurons than astrocytes, with ∼15% of active cells being SR101 positive. The ability to discriminate between neurons and astrocytes was confirmed by the patch-clamp results of single neurons.

The slices were placed in a recording chamber on a moveable bridge platform (Luigs and Neumann) and perfused with aCSF at ∼1 ml/min. Fluorescence imaging was conducted using a Nikon FN1 upright microscope with LED-based illumination (Cairn Research) and acquired using a Hamamatsu ORCA-ER camera. Contrast and brightness were also adjusted to enhance morphological details. In these experiments, acquisition was controlled with Hamamatsu Simple PCI software. Images were typically acquired at 4 Hz. Based on analysis of data acquired from recording SIC-associated calcium elevations in single patch-clamped neurons, calcium elevations were accepted as slow calcium responses (SCRs) if the total duration (10% rise to 90% decay) was between 5 and 30 s and if Δ*F* was >;3%. Acceptance criteria for SCRs in different neurons being synchronized was that 10% rise time (i.e., SCR initiation) occurred within a 2 s window. Imaging experiments were conducted at 32°C.

##### Preexposure experiments.

After a 1 h recovery period in the maintaining aCSF, 200 μm glutamate (Pre-Glut) or D-Asp (Pre-D-Asp) was added as indicated in the text. The aCSF solution also contained a 2 mm concentration of the Glut receptor antagonist kynurenic acid to prevent Glut-receptor-mediated excitotoxicity. Slices for control experiments were maintained in a solution containing kynurenic acid without Glut or D-Asp. Slices were maintained in the preexposure solution until recording (range 1.5–6 h unless specifically stated). For experiments, slices were removed from the pretreatment solution and perfused with aCSF for 15 min to allow washout of kynurenic acid. Experiments were conducted in aCSF not containing Glut or D-Asp.

##### Electrophysiology.

Patch-clamp recordings were made using borosilicate pipettes (Harvard Instruments; 2–4 MΩ) containing an internal solution containing the following (in mm): KMeSO_4_ 120, HEPES 10, EGTA 0.1, Na_2_ATP 4, and GTP 0.5 with osmolarity adjusted to 295 mOsm with KCl. For combined electrophysiological and imaging experiments, EGTA was replaced with penta-potassium Fluo-4 100 μm. Currents were recorded using a Multiclamp 700B amplifier and data were acquired and analyzed using PClamp 9 (Molecular Devices). SICs were discriminated from possible EPSPs by only accepting events as SICs that had a time to peak of >;20 ms and an amplitude of >;20 pA, as described previously (Pirttimaki et al., 2011, Pirttimaki and Parri, 2012). Synaptic stimulation was conducted using a Multichannel systems STG 1002 stimulator with bipolar electrode. A 100 μS stimulus eliciting half-maximal postsynaptic response was used.

##### Random response model.

To provide a comparison of the expected number of synchronized events that would arise by chance, a computer-based random number generator was used that generated events for individual “neuronal” elements. The model parameters were based on data from experiments of emerging calcium events in TTX and consisted of 20 cells run for 120 s each with a 0.008 s^−1^ (0.50 min^−1^) chance of generating a response. Synchrony was calculated as for brain slices.

##### Statistics.

All quantitative data in the bar graphs are presented as mean ± SEM. Comparison of two independent groups was performed with a two-tailed Student's *t* test; more than two groups were analyzed with ANOVA with *post hoc* Bonferroni method where indicated. Analysis of distribution was performed with the Kolmogorov–Smirnov (KS) test. Significance depicted in figures was: *p < 0.05, **p < 0.01, ***p < 0.005.

## Results

### Glut GT release is dependent on uptake

**Figure 1.**
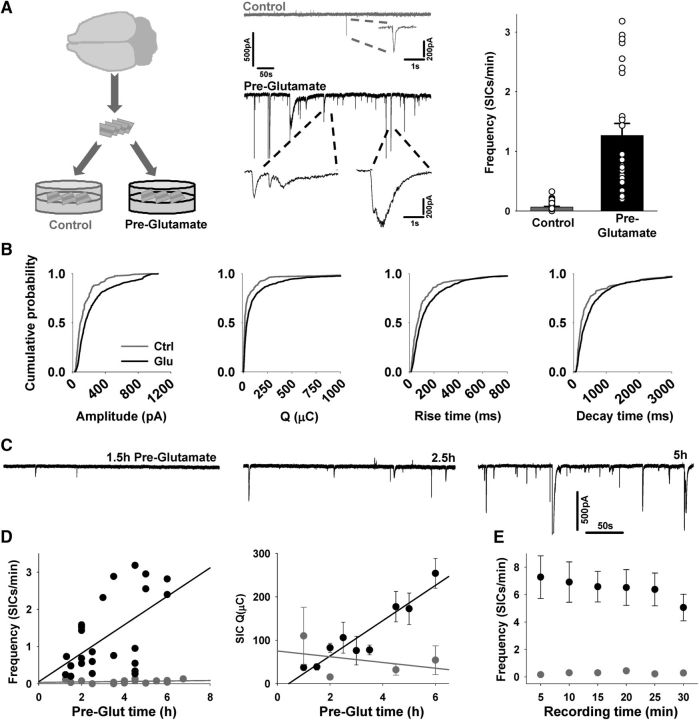
Preexposure of acute brain slice to Glut increases SIC emergence. ***A***, Left diagram, Brain slices were cut and placed in control aCSF (gray dish) or in a Glut preexposure aCSF (black dish). Top middle trace (gray) from a VB TC neuron in a slice stored in control aCSF exhibiting a single SIC. Bottom (black) trace is a patch-clamp recording from a TC neuron in a slice preexposed to aCSF containing 200 μm Glut for 4 h, exhibiting a large number of SICs. Bar graph on the right illustrates SIC frequency from a number of experiments. White circles indicate values from individual recordings for the two conditions. ***B***, Cumulative probability curves for SIC amplitude, charge, rise times, and decay times for control (gray plots) and >;2 h Pre-Glut treatment (black). ***C***, Example traces from three different cells preexposed to Glut containing aCSF for the indicated times before recording. ***D***, Plot from a number of experiments of SIC frequency against preexposure time for control aCSF (gray symbols) and Glut-containing aCSF (black symbols) fitted with regression lines (left) and variation of mean charge (Q, right) with pretreatment times for control (gray lines and symbols) and Glut (black lines and symbols) containing aCSF. ***E***, Mean SIC frequency at different time points during recording for cells from the two groups showing that increased SIC frequency in Glut preexposed slices is sustained. All recordings were conducted in the presence of TTX.

Although astrocytes have been shown to release Glut as a GT, they are thought not to synthesize this amino acid, but rather to uptake Glut after neuronal synaptic release and convert it to glutamine, which is then transported and cycled back to the presynapse (Hertz et al., 1999). We therefore reasoned that Glut released from astrocytes must first be taken up via EAATs. After cutting, slices were either maintained for a period (>;1 h) in a control storage aCSF or in an aCSF containing Glut (200 μm; Fig. 1*A*). Recording from neurons in Glut preexposed (Pre-Glut) VB thalamus slices revealed a substantial increase in SIC frequency (control SIC frequency: 0.07 ± 0.01 /min; Pre-Glut: 1.26 ± 0.2 SICs/min; Student's *t* test *p* < 0.0001; *n* = 50, 25 cells respectively; Fig. 1*A*). SIC amplitude and kinetic parameters were different in Glut preexposed slices compared with control: amplitude: control 140.5 ± 12.6 pA, Pre-Glut 243 ± 7.4. pA; charge: control:328.6 ± 265 μC, Pre-Glut 136.45 ± 10.85 μC; rise time: control 165.4 ± 40.5 ms, Pre-Glut 172.4 ± 7.8 ms; decay: control 1316.7 ± 668.8 ms, Pre-Glut 729.8 ± 40.8 ms (control *n* = 133 SICs, Pre-Glut *n* = 948 SICs), all cumulative distribution comparisons *p* < 0.0001, KS (Fig. 1*B*). Despite rightward shifts in the cumulative distributions, mean values for charge and decay times apparently decrease after pretreatment. This is likely due to an increase in midrange values in the SIC amplitude distribution because inspection of median values confirm an increase in SIC charge and decay time (decay: control: median 231.6 ms, Pre-Glut median 366.6 ms; charge: control 164.4 μC, Pre-Glut 376.83 μC).

SIC frequency increased with Glut preexposure in a time-dependent manner (Fig. 1*C*; *r*^2^ = 0.3, Pearson's correlation *p* < 0.005; *n* = 25 cells; Fig. 1*D*). SIC frequency in slices exposed for <2 h was 0.43 ± 0.1/min, whereas after incubating for 5–6 h, it was 2.68 ± 0.1/min (Student's *t* test *p* < 0.001). There was also a significant time-dependent change in SIC properties (charge Q *r*^2^ = 0.9, Pearson's correlation *p* = 0.0002; rise time *r*^2^ = 0.85, *p* = 0.0004; decay time *r*^2^ = 0.8, *p* < 0.005; Fig. 1*D*). In contrast, slices maintained in control aCSF showed no time-dependent change in SIC frequency (*r*^2^ = 0.06, *p* = 0.4; *n* = 15 cells; Fig. 1*D*), nor in SIC parameters with time (control amplitude *r*^2^ = 0.1, *p* = 0.6; rise time *r*^2^ = 0.06, *p* = 0.8; charge *r*^2^ = 0.06, *p* = 0.6; decay time *r*^2^ = 0.2, *p* = 0.6). The effect of Glut pretreatment was not confined to a period when somatosensory developmental changes were occurring, but were also seen in slices from ages P19–P21 (control: 0.14 ± 0.1 SICs/min, *n* = 16 cells; Pre-Glut: 0.56 ± 0.16 SICs/min, *n* = 10 cells, *p* < 0.05). This is consistent with our previous work on mGluRI-mediated SIC frequency increase (Pirttimaki et al., 2011) indicating that SIC frequency can be regulated at older than 3 weeks. The existence of an elevated SIC frequency in recordings from preexposed slices after their removal from Glut-containing solutions indicated that the continued presence of extracellular Glut was not necessary to sustain SIC generation. We investigated the robustness of this sustainment. SIC frequency from control slices (*n* = 50 cells) were compared with SIC frequency from preexposed slices (*n* = 25) in 5 min intervals over a 30 min recording period (Fig. 1*E*). Glut pretreatment induced SIC frequency was sustained throughout the recording period (Fig. 1*E*), therefore indicating a long-lasting sustained increase in SIC frequency dependent on previous GT supply. There was no time-dependent difference in SIC frequency for either control or preexposed slices (ANOVA). However, pooled and SIC rates were significantly higher in preexposed slices (*p* < 0.001, Student's *t* test) compared with control.

**Figure 2.**
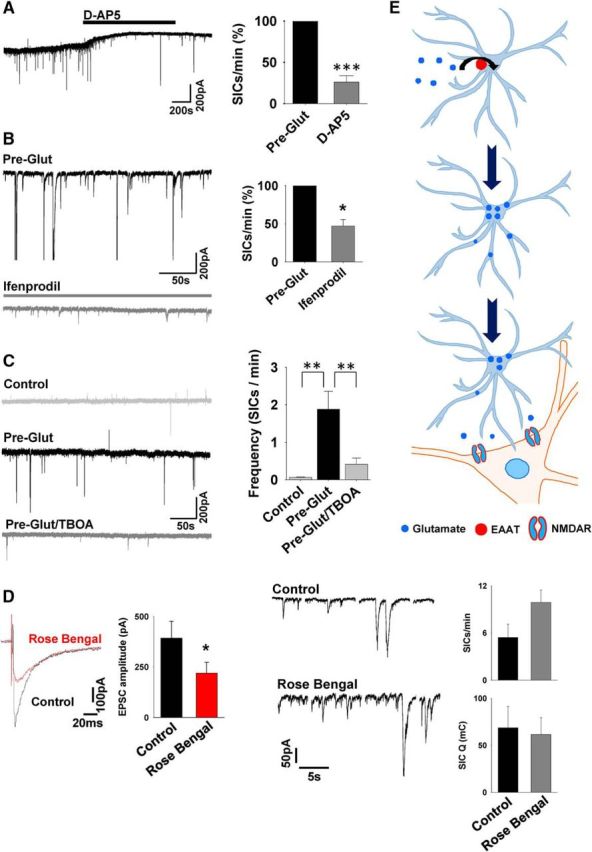
Increased SIC frequency is EAAT dependent. ***A***, TC neuron recording after Glut preexposure showing the blocking effect of the NMDAR antagonist D-AP5 on SICs. Summary data from multiple experiments are shown in the bar graph on the right. ***B***, Experiment illustrating that the NR2B selective antagonist ifenprodil (gray trace, same cell) inhibits SICs. Summary data from multiple experiments are shown in the bar graph on the right. ***C***, Example traces from recordings in slices preexposed in control aCSF (gray) with Glut for 2 h (black), and with Glut in the presence of the EAAT blocker TBOA for 4 h (light gray). Bar graph on the right summarizes a number of experiments. ***D***, Traces (left) show evoked postsynaptic current before (black) and 20 min after perfusion of the VGlut inhibitor Rose Bengal (red) in a slice after reexposure to Glut for 2 h. Bar graph summarizes data from four experiments. Traces on right show SICs from the same neuron in the two conditions (evoked PSCs omitted for clarity) after a 3 h Pre-Glut. Bar graphs on the right display SIC frequency and charge in control and with Rose Bengal. ***E***, Illustration depicting data interpretation that Glut is taken up via EAAT transporters and later released to activate extrasynaptic NMDARs on the neuron.

SICs from Glut preexposed slices exhibited similar signaling properties to those of SICs in control conditions and as described previously (Pirttimaki et al., 2011, Pirttimaki and Parri, 2012). SICs were insensitive to TTX (1 μm; Pre-Glut: 0.85 ± 0.24; Pre-Glut TTX 0.69 ± 0.17 SICs/min; paired Student's *t* test *p* = 0.3; *n* = 5 cells). SICs were reversibly inhibited by the NMDAR antagonist D-AP5 (50 μm; Fig. 2*A*): Pre-Glut: 1.3 ± 0.5 SICs/min, D-AP5: 0.37 ± 0.15 SICs/min (paired Student's *t* test *p* < 0.0005; *n* = 6 cells). The remnant SICs during D-AP5 application were reduced significantly in amplitude compared with those in control conditions (Pre-Glut: 231.0 ± 14.7 pA, *n* = 227; D-AP5 142.1 ± 27.3 pA, *n* = 40 SICs; Student's *t* test *p* = 0.01). Similarly, in the presence of the NR2B subunit containing NMDAR antagonist ifenprodil (10 μm), frequency was reduced to 47.2 ± 8.5% of control (Pre-Glut: 1.83 ± 0.87 SICs/min; ifenprodil: 0.8 ± 0.4 SICs/min; paired Student's *t* test *p* < 0.05; *n* = 3 cells; Fig. 2*B*) and the remaining SICs were 64.4% smaller in amplitude (Student's *t* test *p* < 0.05). In some Pre-Glut experiments, application of D-AP5 resulted in a baseline shift indicative of a block of tonic current (Fig. 2*A*). An increase in membrane noise was also seen in Pre-Glut experiments compared with control, which was consistently reduced by D-AP5 (*n* = 6, data not shown). These results suggest that tonic Glut may result from Glut preexposure in addition to phasic Glut release. It has been shown that tonic Glut is also astrocyte dependent (Cavelier and Attwell, 2005, Le Meur et al., 2007).

If astrocytes release Glut after uptake via EAATs, then the increased frequency in SICs that we observed after preexposure would be abrogated by EAAT inhibition. We tested this hypothesis by including the broad-spectrum EAAT inhibitor TBOA (300 μm) in the preexposure medium together with Glut. In these experiments, TBOA indeed attenuated the Glut preexposure-induced increase in SIC frequency significantly (control: 0.07 ± 0.01 SICs/min, *n* = 15 cells; Pre-Glut: 1.87 ± 0.5 SICs/min, *n* = 6; Pre-Glut/TBOA: 0.41 ± 0.17 SICs/min *n* = 7cells). Statistical analysis indicated an increased SIC frequency in Pre-Glut compared with control (*p* < 0.01 ANOVA and Bonferroni method), whereas there was no significant difference between control and Pre-Glut/TBOA (ANOVA and Bonferroni method), indicating a central role for EAAT uptake in supply-enhanced SIC generation (Fig. 2*C*). Mean amplitude was also significantly lower in Pre-Glut/TBOA preexposed slices (Pre-Glut: 268.4 ± 12.3 pA, Pre-Glut/TBOA: 154.3 ± 19.2 pA; Student's *t* test *p* < 0.0005; *n* = 334, 76 SICs respectively). Presynaptic endings may also express EAATs, which could uptake Glut during the pretreatment exposure and increase the amplitude of EPSCs that may be mistaken during analysis for SICs. We used the vesicular Glut transporter (VGluT) inhibitor Rose Bengal to test this hypothesis. After Glut pretreatment, SICs and evoked postsynaptic currents were recorded and the effect of Rose Bengal (30 μm) determined (Neale et al., 2013, Neale et al., 2014). Synaptic currents were significantly reduced by Rose Bengal (control: 392 ± 83.68 pA, Rose Bengal 219.92 ± 52.34 pA, *p* < 0.05, *n* = 5 neurons), whereas SIC frequency was not reduced (control: 4.33 ± 1.08 SICs/min, Rose Bengal 10.1 ± 1.05 SICs/min), and SIC charge was unaffected (control: 68.61 ± 22.68 mC, Rose Bengal 61.62 ± 17.84 mC; Fig. 2*D*). Membrane noise was seen to increase after Rose Bengal treatment; however, it is not clear whether this is due to an effect on tonic Glut current. Together, these data indicate that the Glut-preexposure-enhanced GT-mediated SICs are dependent on EAAT uptake and are mediated by extrasynaptic NMDARs (Fig. 2*E*). Therefore, they act and behave in the same way as spontaneously recorded SICs in VB thalamus (Parri et al., 2001, Pirttimaki et al., 2011, Pirttimaki and Parri, 2012).

### EAAT substrate D-Asp is released as a false GT

**Figure 3.**
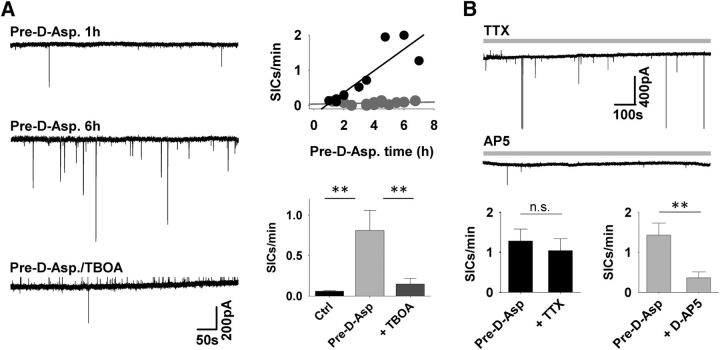
D-Asp can be used as a false GT. ***A***, Example recordings from TC neurons in slices after 1 h (top) and 6 h (middle) preexposure to aCSF containing 200 μm D-Asp and preexposed to D-Asp in the presence of the EAAT blocker TBOA for 3.5 h (bottom). Scatter plot shows the dependence of SIC frequency on the duration of D-Asp pretreatment time for (control: gray symbols, Pre-D-Asp: black symbols. Bar graph illustrates comparison SIC frequency in different preexposure conditions. ***B***, Example recordings from TC neurons in slices after 6.5 h D-Asp preexposure in the presence of TTX and after the application of D-AP5. Data are summarized for the different conditions in the bar graph below.

It is known that certain neurotransmitter molecules can be introduced into neurons where they are not normally present and that they will be released as false neurotransmitters (Dan et al., 1994). The identified central role of EAATs in Glut-enhanced SICs therefore suggested that other EAAT substrates might potentially be used as “false GTs.” To test this, we selected the amino acid D-aspartic acid (Danbolt, 2001, D'Aniello, 2007) because, interestingly, it is a selective agonist at NMDARs (Verdoorn and Dingledine, 1988), so, if released, should also elicit SICs. Indeed, this proved to be the case, with SIC frequency in D-Asp (200 μm) preexposed slices being 0.81 ± 0.25 SICs/min (*n* = 10 cells) compared with 0.06 ± 0.01 SICs/min (*n* = 15 cells) in control (Fig. 3*A*). Furthermore, the results showed a preexposure time-dependent increase in SIC frequency similar to that seen with Glut (*r*^2^ = 0.76, Pearson's correlation *p* < 0.005; Fig. 3*A*). A preexposure of 2 h or less resulted in a frequency of 0.14 ± 0.02 SICs/min (*n* = 3 cells) compared with 1.28 ± 0.3 SICs/min (*n* = 5) cells with preexposure longer than 3 h. The Pre-D-Asp-induced increase in SIC frequency was significantly higher than control slices (*p* < 0.01 Bonferroni method; Fig. 3*A*), but not different from Glut preexposure increase. TBOA (300 μm) inhibited the Pre-D-Asp-induced increase in SIC frequency (Pre-D-Asp/TBOA: 0.15 ± 0.07 SICs/min; *n* = 6 cells, *p* < 0.01, Bonferroni method; Fig. 3*A*).

As expected, blocking neuronal activity with TTX (1 μm) did not affect the frequency of D-Asp SICs (Pre-D-Asp 1.28 ± 0.3 SICs/min, Pre-D-Asp/TTX: 1.04 ± 0.3 SICs/min; paired Student's *t* test *p* = 0.3; *n* = 5 slices; Fig. 3*B*). Frequency was reduced from 1.43 ± 0.3 SICs/min to 0.36 ± 0.15 SICs/min in presence of D-AP5 (50 μm; paired Student's *t* test *p* < 0.05; *n* = 4). SICs recorded from Pre-D-Asp-treated slices displayed similar kinetic properties to Pre-Glut slices (Pre-D-Asp: amplitude 255.8 ± 18.0 pA, rise time 156.2 ± 11.9 ms, Q 116.2 ± 16.7 ms, *p* = 0.3 compared with Pre-Glut, *n* = 10 cells, 183 SICs). This demonstrates that Pre-D-Asp leads to SICs with similar properties to normal spontaneous SICs and those arising after Glut preexposure, consistent with a common mechanism. Furthermore, the data show that preexposure to D-Asp can be used as a potential tool to investigate selective functional overexpression of GT release and selective NMDAR-mediated effects.

### SIC frequency increase is independent of mGluRs

**Figure 4.**
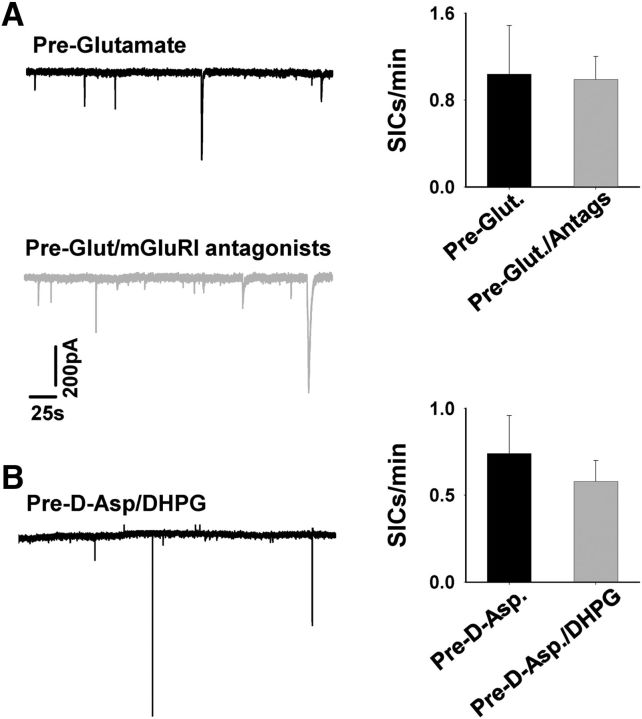
Enhanced GT-mediated SIC frequency is not mGluRI dependent. ***A***, Example recording from two different neurons after Glut pretreatment for 2.5 h (black trace) and after pretreatment with Glut and mGluRI antagonists for 3 h. Bar graph summarizes SIC frequencies in the different conditions from a number of neurons. ***B***, Recording from a neuron after pretreatment with D-Asp and mGluRI agonist DHPG for 4.5 h. Bar graph summarizes SIC frequencies in the different conditions from a number of neurons. Recordings were conducted in the presence of TTX.

We have previously identified an enhancement of SIC frequency induced by the action of synaptically released Glut via mGluR group I receptors (mGluRIs). To determine whether this pathway contributed to or could modify the observed effect of preexposure, slices were exposed to Glut in the presence of the mGluR_5_ antagonist MTEP (20 μm) and the mGluR_1_ antagonist (s)-4-CPG (100–200 μm). The presence of mGluR group I antagonists did not affect the degree of induced Glut SIC enhancement (Pre-Glut: 1.04 ± 0.45 SICs/min, Pre-Glut + MTEP + (s)-4-CPG: 0.99 ± 0.2 SICs/min; Student's *t* test *p* = 0.9; *n* = 5, 6 cells respectively), nor affected SIC amplitude (Pre-Glut: 267.9 ± 20.9 pA in Glut preexposed slices and 252.4 ± 17.7 pA in Pre-Glut with mGluRI antagonists, Student's *t* test *p* = 0.6; *n* = 155 and 192 SICs, respectively; Fig. 4*A*), indicating that mGluRI activation is not necessary for the enhancement of SIC frequency seen in preexposure experiments.

In the converse experiment, we tested the ability of mGluRI activation in further enhancing SIC frequency in D-Asp preexposed slices. Mean frequency after D-Asp and DHPG (100 μm) preexposure was 0.56 ± 0.11 SICs/min (*n* = 5 cells) compared with 0.74 ± 0.23 SICs/min with D-Asp alone (*n* = 10; Student's *t* test *p* = 0.6; Fig. 4*B*). The results indicate that the enhanced SIC frequency caused by preexposure is independent of mGluRI activation and that the effect of mGluRI activation is not additive to preexposure, suggesting that preexposure saturates the GT release mechanism.

### SICs generate neuronal SCRs

**Figure 5.**
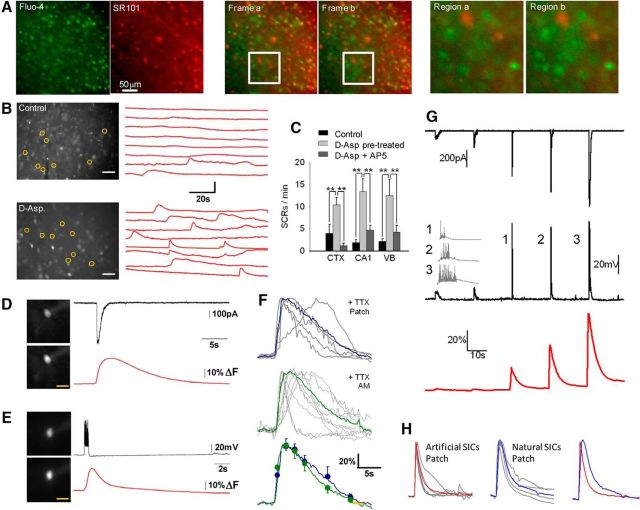
SIC-induced neuronal calcium signaling. ***A***, Fluorescence image of a cortical slice loaded with Fluo-4AM (left) and SR101. Middle, Overlaid green and red images show the area at a time at beginning of acquisition (Frame a) and during a synchronized event (Frame b), with square bounded region delineating an area exhibiting a calcium increase in cells not SR101 loaded. Right, Active regions expanded. ***B***, Fluorescence image of a field of neurons in a cortical slice loaded with Fluo-4 AM (left) and traces of fluorescence over time with spontaneous SCRs (right) for circled neurons in the presence of TTX. Scale bar, 50 μm. Top, Data from a slice preexposed in control aCSF. Bottom, Slice preexposed with D-Asp. ***C***, Summary data of SCR frequency in control and D-Asp preexposed slices from barrel cortex, CA1 hippocampus, and VB thalamus illustrating their sensitivity to AP5. ***D***, Patch-clamped neuron in the presence of TTX with pipette containing Fluo-4. The images on the left illustrate fluorescence before and at peak of the SCR. Traces show a spontaneous recorded SIC (top) and elicited fluorescence SCR (bottom). ***E***, Patched neuron with pipette containing Fluo-4 recorded in current-clamp mode. Traces show a spontaneous burst (top) and elicited fluorescence calcium signal (bottom). ***F***, Spontaneous SCRs from single-neuron patch-clamp recordings (top, blue traces) normalized to amplitude to illustrate kinetics. Middle traces (green) show example spontaneous SCRs from Fluo-4 AM-loaded slices in TTX. Gray lines represent individual SCRs; colored lines, the mean. Bottom traces show the mean traces from the two conditions superimposed with symbols illustrating SEM. ***G***, Top trace shows responses in a patch-clamped neuron to injection of recorded SIC waveforms. Middle trace shows a neuron response to the SICs in current-clamp mode, with expanded action potentials in numbered responses to the left. Red bottom trace shows fluorescence changes during the SIC evoked events. ***H***, Nest of calcium responses normalized to peak amplitude generated by injected SICs (red) and generated by spontaneous SICs (blue) with averaged comparison to the right.

We sought to determine the influence of phasic astrocyte GT events on neuronal activity by investigating how many neurons an astrocytic GT event acting at neuronal NMDARs could synchronize. D-Asp preexposed slices were loaded with the calcium indicator dye Fluo-4 and imaged over time. Wide-field LED excitation enabled the imaging of large neuronal populations at a frequency of 4 Hz (Fig. 5*A*). In preexposed slices, there was a marked increase in the emergence of neuronal calcium elevations that were observed in the presence of TTX (Fig. 5*B*,*C*) and blocked by AP5 (Fig. 5*C*). Calcium elevations in somatosensory cortex, CA1 hippocampus, and VB thalamus shared these characteristics (Fig. 5*C*; cortex after preexposure: 0.46 ± 0.09, D-AP5: 0.11 ± 0.02; CA1 after preexposure: 0.56 ± 0.08, D-AP5: 0.20 ± 0.05; VB after preexposure: 0.53 ± 0.09, D-AP5: 0.18 ± 0.04 SICs/min; cortex: *p* < 0.0001; CA1: *p* < 0.001; VB: *p* < 0.01, ANOVA with Bonferroni method). To confirm that these were indeed due to SIC-mediated events, single-cell patch-clamp recordings were conducted with a pipette containing Fluo4. This enabled the direct identification and analysis of SIC-induced calcium elevations (Fig. 5*D*,*E*). Comparison of the profiles of SIC-induced single-cell calcium responses in D-Asp preexposed slices with elevations seen in slices in the presence of TTX showed that the kinetics were indistinguishable (Fig. 5*F*). As further verification of our ability to identify the distinct profile of SIC-induced neuronal calcium elevations, we compared SIC-induced calcium elevations with those induced by injecting recorded SIC waveforms in the same neurons (Fig. 5*G*). Although such waveforms mimic the effect of SICs on membrane potential (Pirttimaki et al., 2011), the calcium elevations elicited by injected waveforms that were not NMDAR mediated had faster 10–90% rise times (injected: 0.4 ± 0.06 s, naturally occurring:0.89 ± 0.15 s) and faster 90-10% decay (injected: 3.32 ± 0.82 s, emerging: 8.66 ± 2.43 s, *n* = 5 cells; Fig. 5*H*) than emerging NMDAR-mediated SCRs occurring in the same cells, thus confirming the distinctive kinetic properties of SIC-induced calcium elevations. As a correlate to SICs, identified neuronal responses were therefore termed SCRs.

### False GT release synchronizes neuronal groups

**Figure 6.**
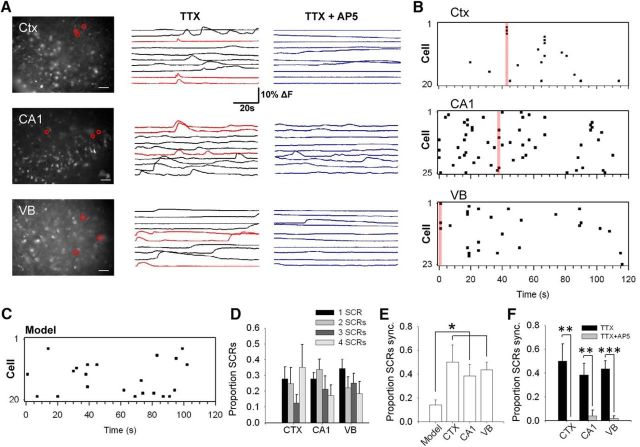
Astrocyte-induced synchronized neuronal responses. ***A***, Fluorescence images of Fluo-4 loaded slices of cortex, CA1 hippocampus, and VB thalamus. Neurons exhibiting calcium elevations are circled. Fluorescence over time traces for the neurons are displayed on the right in the presence of TTX and after the addition of AP5. Traces in red are those exhibiting a synchronized elevation in red circled cells in image. Slices were preexposed with D-Asp for 2-4 h ***B***. Raster plots of SCRs recorded in neurons from the slices in ***A*** in the presence of TTX. Individual SCRs are denoted as points. Activated neurons are numbered vertically on the *y*-axis. The red bars indicate the example synchronous responses from ***A***. ***C***, Raster plot generated using the simulated probability model for 20 neurons. ***D***, Bar graphs displaying relative proportion of synchronized events composed of different neuron numbers in slices from the different brain areas. ***E***, Bar graph showing comparison of proportion of synchronous neuron groups of three neurons or more compared with the simulated probability model of random release with the same SCR frequency. ***F***, Abrogating effect of AP5 on neuronal synchronization.

Having confirmed the ability to identify the observed SCRs evoked by SICs, we then investigated synchronized neuronal events in different brain areas. To reduce the possibility of false-positive or spurious reporting, events were considered synchronized if they had reached threshold within a restricted detection window of 2 s. Synchrony was measured as the proportion of total SCRs recorded that occurred in events in which at least three neurons were synchronous. Events in which only two neurons were synchronous were discounted to minimize the risk of false-positives. Imaging was conducted in slices from somatosensory cortex, CA1 hippocampus, and VB thalamus (Fig. 6*A*). To enable comparison with events arising randomly, we used a “random response model” simulation exhibiting events of the same frequency range (Fig. 6*C*). Analysis revealed that groups of neurons exhibited synchronized SCRs in slices from all imaged regions (Fig. 6*C*). The relative proportion of SCRs in synchronized events with 3 or more neurons was increased significantly in Pre-D-Asp slices compared with control (cortex: 0.5 ± 0.14, *n* = 7, control: 0.026 ± 0.028, *n* = 11, *p* < 0.05. CA1: 0.38 ± 0.097, *n* = 7, control: 0.042 ± 0.046, *n* = 7, *p* < 0.01. VB: 0.43 ± 0.067, *n* = 7, control: 0.037 ± 0.04, *n* = 8, *p* < 0.01, *t* test) and significantly greater than predicted from the random response model, in which the proportion of synchronous SCRs was 0.13 ± 0.04 (*n* = 15; by ANOVA with Bonferroni method: cortex, *p* < 0.005; CA1, *p* < 0.05; VB, *p* < 0.05; Fig. 6*D*,*E*). The incidence of synchronized neuronal groups was abolished in the presence of AP5 (cortex: *p* < 0.01, CA1: *p* < 0.005, VB: *p* < 0.0005, *t* test; Fig. 6*F*), consistent with an NMDAR SIC-mediated synchronization mechanism.

**Figure 7.**
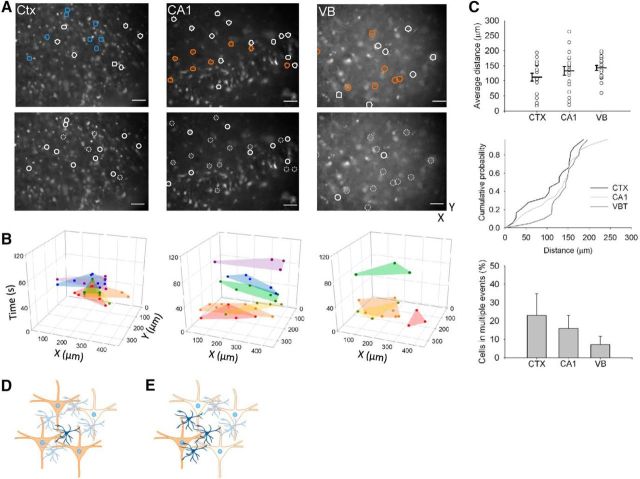
Spatial extent of synchronized neuronal activity. ***A***, Images of Fluo-4-AM-loaded slices from cortex, CA1 hippocampus, and VB thalamus. Same colored circles designate neurons that displayed event synchronization. Top images indicate two examplar synchronized groups for each area. Bottom images indicate for these exemplar cells those that exhibit synchronized elevations (circled) and neurons that participate in multiple synchronized groups (dotted circles) Scale bar, 50 μm. ***B***, 3D plots with colored circles indicating synchronized neurons. Shading illustrates area encompassed by events (*x–y*) and vertical axis *z* shows the emergence of synchronized neurons and slice areas over time. ***C***, Plots of average distance between neurons in a synchronized group in the different brain regions. Cumulative probability plot (right) of distance between synchronized neurons. Bottom, Bar graph of relative proportion of neurons in different brain areas participating in multiple synchronized events during the recording period. ***D***, Interpretation of synchronization data: a single astrocyte (dark blue) releases GT, which activates and so synchronizes multiple neurons (dark orange). ***E***, Depiction of “hub” neuron data interpretation: a single neuron can be activated by GT release from multiple astrocytes, each of which activate a different complement of the local neuronal population.

Different brain areas have different neuronal and astrocytic architecture (Houades et al., 2006). Astrocytes releasing GT to excite the dendrites of multiple neurons might therefore have different degrees of spatial and network influence depending on whether the excited dendrites extended diffusely and relatively far from the cell somas or if the cells were locally compact. We therefore analyzed the spatial extent of synchronized neurons in slices from cortex, hippocampus, and VB thalamus (Fig. 7*A*). Analysis of synchronized neuronal groups revealed neurons delineating overlapping areas (Fig. 7*B*), with different groups displaying activity over time. The range of distances between synchronized neurons in the three imaged regions ranged between 18 and 263 μm, with the mean distances being similar (cortex: 112 ± 13.2, *n* = 18 areas. CA1: 133 ± 14, *n* = 20 areas. VB: 142 ± 7.9, *n* = 20 areas, *p* = 0.19, ANOVA; Fig. 7*C*). There was also no significant difference between the population distributions of the distances between synchronized neurons in the different brain areas (*p* >; 0.05, KS). Neurons in all brain areas participated in synchronized events with different groups of neurons over time. The cortex seemed to have the greatest relative proportion (cortex: 0.23 ± 0.12, *n* = 7 slices; CA1: 0.16 ± 0.07, *n* = 8 slices; VB: 0.07 ± 0.046, *n* = 7 slices; Fig. 7*C*). However, the apparent difference was not significant (*p* = 0.38, ANOVA). The results showing that neurons are synchronized by NMDAR-mediated SICs in all brain areas tested support the notion that astrocytes release GT that acts at multiple local neurons (Fig. 7*D*), whereas neurons that participate in multiple synchronized neuronal groups indicate an astrocyte–neuron interaction in which multiple astrocytes can release GT that affects an individual neuron (Fig. 7*E*).

## Discussion

In this study, we found that astrocyte release of Glut and D-Asp is supply dependent and that the release of these amino acid GTs can be upregulated chronically by preexposure. We also found that preexposure with an EAAT substrate increased GT release, showing that EAAT substrate specificity is central to the mechanism and illustrating the principle that a false GT can be taken up by astrocytes and released. Using D-Asp, a selective NMDAR agonist, as a false GT, we were able to investigate astrocyte–neuron NMDAR-mediated synchronization in somatosensory cortex, CA1 hippocampus, and VB thalamus.

Astrocytes take up synaptically released Glut via EAATs. This is converted to glutamine by the action of glutamine synthase and cycled back presynaptically, where it is converted to Glut (Hertz et al., 1999). Our findings that exposure of acute brain slices to increased extracellular Glut increases frequency and individual SIC charge recorded in neighboring neurons agree with previous data showing increased astrocyte Glut release by glutamine synthase inhibition (Jabaudon et al., 1999, Cavelier and Attwell, 2005) and of “Glut induced exocytosis” (Xu et al., 2007) recorded after focal delivery of high-concentration Glut to astrocytes. Increased SIC frequency with time was abrogated by blocking EAATs during Glut preexposure, indicating a pivotal and specific role for these transporters in the supply-dependent increase in GT release.

Our selection of D-Asp as a false GT candidate was governed by its characteristic of being a substrate of EAATs and its selective agonist effect at NMDARs. Importantly, we found that preexposure to D-Asp mimicked the effects seen with Glut, indicating a likely common mechanism for astrocyte–neuron Glut and D-Asp signaling.

D-Asp is not a transmitter that is completely alien to the brain. There is expression during development that is highest in the prenatal period, falls after birth, and is almost completely absent by postnatal week 4 (Errico et al., 2015), although it persists in dentate gyrus, where it may be involved in continuing neurogenesis (Kim et al., 2010). However, even when present developmentally, its cellular distribution is confined to neurons and it is not present in astrocytes (Schell et al., 1997). Some studies have suggested continuing physiological roles for D-Asp, but a study on synaptic transmission in the hippocampus concluded that Asp levels were too low to have any physiological impact (Herring et al., 2015). These characteristics therefore support the designation and utilization of D-Asp as a false GT in this study.

The precise mechanism of release and its control after uptake is, however, unclear. Whereas D-Asp is a substrate for cytoplasmic membrane EAATs, it is not a substrate for the vesicular transporter VGluT (Tabb and Ueda, 1991), which would be required for vesicular packaging. Although this might suggest that D-Asp is not released via a vesicular mechanism, it is possible that transport occurs via another system (Nadler, 2011). It is also interesting that increased preexposure leads to increased SIC event charge, indicative of an increase in the amount of GT released during single events in addition to an increased frequency of release of GT “packages.” Whether this is due to an increase in GT content of already occurring subthreshold events or if pretreatment actually leads to an increase in release events is not clear.

There is precedence in the use of false transmitters to investigate transmitter release in both neuronal and non-neuronal cells (Dan and Poo, 1992) found that introducing acetylcholine (ACh) into glutamatergic hippocampal pyramidal neurons resulted in phasic release events that triggered inward currents in reporter cells. A similar study on fibroblasts involving injection and pretreatment with ACh (Chang et al., 1998) concluded that ACh false transmitter was taken up via an endocytic pathway and phasic release was a result of the endocytic vesicular recycling. Our experimental results show partial similarities with the results of this study; however, importantly, uptake is not via a nonspecific endocytotic route but rather via specific transporters indicative of inclusion of the exogenous false transmitter D-Asp in a regulated signaling mechanism. However, our data do suggest that astrocytes and neurons (Dan et al., 1994) are able to package multiple transmitters if they are present at sufficient levels in the cytoplasm.

We found that enhanced GT release was not affected by mGluRI group antagonists and that D-Asp-enhanced false GT release was not further enhanced by mGluR group I agonists. This indicates that the supply-dependent increase in GT release is distinct from the mGluR-dependent increase that we have described previously (Pirttimaki et al., 2011) and so represents another distinct mechanism of GT release emergence. The lack of effect of additional mGluR activation on D-Asp release likely indicates that GT release may be modulated by multiple mechanisms, but that the sum exerted modulation lies within a finite range. This may also indicate that, under some circumstances, increased supply could override physiological control, which may be relevant for pathophysiological conditions in which a large amount of extracellular Glut is acutely available, as may occur in epilepsy or Alzheimer's disease (Talantova et al., 2013, Soukupova et al., 2015).

Using D-Asp as a false GT enabled us to probe selectively the astrocyte-mediated activation of neuronal NMDARs and to determine the effect of this specific mechanism on the extent and emergence of neuronal synchronization. Potentially unphysiological calcium elevation stimulation methods are unnecessary (Hamilton and Attwell, 2010, Wang et al., 2013), so the false GT enables the functional upregulation of GT release as an increased observation of events. Our data therefore relate astrocyte GT release to the emergence of activated neuronal groups and the monitoring of how groups are recruited over time.

Analyses of detected synchronized neuronal events in somatosensory cortex, CA1 hippocampus, and VB thalamus revealed functional excitatory connectivity between astrocytes and neurons that can result in synchronized activation of neurons >;200 μm apart. This exceeds previous reports of synchronization in the hippocampus (Angulo et al., 2004, Fellin et al., 2004), but is consistent with a situation in which astrocytes release GT onto distal dendrites. Our data reveal that emerging astrocyte-generated neuronal synchronization is dynamic and that overlapping neuronal domains emerge over time, indicating a potential for astrocytes to sustain repetitive local neuronal modulation. The finding that the number and extent of activated neurons were conserved between brain regions was somewhat contrary to expectations because there are major anatomical and functional differences between the regions. The VB thalamus does not display a laminar structure, whereas the hippocampus CA1 and barrel cortex do. It has also been reported recently that astrocytes between the areas display heterogeneity in their gap junctional coupling and even protein expression patterns. Astrocytes in the VB thalamus showed low levels of connexin 43 expression and were coupled in pan-glial networks, in which more than half were oligodendrocytes compared with <15% in the hippocampus (Griemsmann et al., 2015). The anatomical architecture of astrocytes is also known to be specialized in different brain regions (Houades et al., 2006); for example, in delineating barrels in the cortex displaying within-barrel preferential coupling (Houades et al., 2008). However, our data show that, despite these anatomical variations, the functional impact of astrocyte synchronization is apparently conserved. Some neurons were seen to participate in more than one synchronized group. The anatomical substrate for this might be that individual neurons can be activated by more than one astrocyte.

### Conclusion

Our results show the potential extent of astrocyte GT-mediated influence in different brain regions and that activated neuronal groups are activated over time. It should be noted that our methods that overexpress GT release functionally using a false transmitter and induce large neuronal currents likely may not reflect functional neuronal firing in physiological conditions, but rather serve to indicate the potential extent of influence and their spatial and temporal relationship. For the NMDAR agonists Glut and D-Asp, the interaction of released excitatory GT with neuronal dendrites would have a depolarizing effect that would affect its responses to synaptic input. The extent of this astrocyte influence might therefore have implications for sensory processing at thalamic and cortical levels. (Roy and Alloway, 1999) and, in all regions, the temporally shifting astrocyte modulation of different local neuronal populations may also be expected to have roles in the manifestation of emergent neuronal network activity. False GTs provide an useful tool with which to investigate GT signaling and their use may also give insight into therapeutic applications.
